# Evaluating Public Perceptions and Attitudes Toward Total Knee Arthroplasty: A Cross-Sectional Study in Saudi Arabia

**DOI:** 10.7759/cureus.48611

**Published:** 2023-11-10

**Authors:** Mashael A Alhussain, Omar A Alrasheed, Hadi A Al Swaidan, Abdullah H Alghamdi, Ibrahim A Al Rajeh, Ali A Alkhamis, Amjaad W Almubarzi

**Affiliations:** 1 General Practice, King Faisal University, Hofuf, SAU; 2 Orthopedic Surgery, King Faisal University, Hofuf, SAU

**Keywords:** chronic knee pain, total knee arthroplasty, knowledge assessment, knee osteoarthritis, public health awareness

## Abstract

Background: Total knee arthroplasty is a surgical procedure used to address knee conditions, including osteoarthritis, that cause persistent pain and impaired joint function. While total knee arthroplasty is effective, misconceptions and knowledge gaps exist among the general public, particularly in Saudi Arabia.

Methods: This cross-sectional study, conducted in Al-Ahsa, Saudi Arabia, utilized an online survey to assess public perception of total knee arthroplasty. A questionnaire was developed, validated, and administered to participants aged 18 and above with a history of chronic knee pain. Data were analyzed for associations between knowledge and perception scores and sociodemographic factors.

Results: Out of 704 participants, the majority exhibited poor knowledge (74.1%) of total knee arthroplasty. Factors influencing knowledge included age, education, income, knowing someone who had total knee arthroplasty, hearing about total knee arthroplasty, receiving total knee arthroplasty information, and awareness of total knee arthroplasty indications. Sociodemographic characteristics such as gender, marital status, and perceptions about total knee arthroplasty prevalence did not significantly impact knowledge scores.

Conclusion: This study reveals a significant knowledge and perception gap among the public in Al-Ahsa, Saudi Arabia, regarding total knee arthroplasty. Tailored education initiatives for patients with chronic knee pain are urgently needed to dispel misconceptions and provide accurate information about total knee arthroplasty. Collaborative efforts between policymakers, healthcare providers, and public health authorities are essential for improving public understanding, enhancing healthcare decision-making, and reducing the burden on healthcare systems.

## Introduction

Total knee arthroplasty is a surgical procedure frequently employed to address persistent and debilitating knee pain and impaired joint function resulting from various underlying knee conditions. While knee osteoarthritis stands as the leading cause of total knee arthroplasty, this surgical intervention is also performed in cases of rheumatoid arthritis, fractures, and tumors [1-3]. total knee arthroplasty was a pioneering medical development, which gained widespread adoption in the 1970s and 1980s. Today, it is widely recognized as a successful intervention for advanced knee arthritis [4]. In patients suffering from symptomatic advanced knee osteoarthritis, total knee arthroplasty is often utilized to alleviate pain and improve functional status [5].

The efficacy of total knee arthroplasty in reducing pain and restoring joint function has been well-documented, contributing to the increasing demand for this surgery [6-8]. Previous research has emphasized that patient preoperative expectations significantly influence total knee arthroplasty outcomes in terms of patient satisfaction and quality of life [9]. Total joint arthroplasties are elective procedures, and understanding long-term patient-centered outcomes can assist individuals in making appropriate decisions when considering future surgery [10]. A cross-sectional study conducted in 2017 in Saudi Arabia revealed a significant misconception about the procedure within the population [1].

Furthermore, previous research delved into the determinants of patient preferences for total knee replacement, specifically comparing African-American and white populations revealed that despite chronic knee pain and radiographic evidence of osteoarthritis in the study samples, they exhibited a reluctance to undergo total knee arthroplasty. These findings underscore the prevalence of misconceptions and knowledge gaps surrounding this surgical procedure among the public [11].

The present study seeks to evaluate the knowledge and attitudes toward total knee arthroplasty among the public in Al-Ahsa, Saudi Arabia. This research endeavors to address the prevailing gaps in public awareness and knowledge of total knee arthroplasty, with the ultimate goal of enhancing patient education, informed decision-making, and healthcare outcomes in the region.

## Materials and methods

Study design

This is a cross-sectional online-survey-based study. An online survey was conducted among the public in Al-Ahsa, Saudi Arabia between March 1, 2023, and May 31, 2023.

Study population

Participants included the adult population in Al-Ahsa, Saudi Arabia, aged 18 and above, with a history of chronic knee pain. Exclusion criteria were participants who were less than 18 years old, had no history of chronic pain, did not belong to the Al-Ahsa population, or those with incomplete responses.

Sample size

The sample size was calculated using the Epi Info software (Centers for Disease Control and Prevention, Atlanta, United States) with the following parameters: a population of 1,300,000 according to the last governmental population census of Al-Ahsa, a 95% confidence level, a response distribution of 50%, and a margin of error of not more than 5%. Based on this, the estimated sample size was 385 participants.

Study instrument

A literature review initially identified pertinent areas of knowledge and perception concerning total knee arthroplasty, and expert input from orthopedics and healthcare specialists refined the questionnaire items. Pretesting with a similar demographic group facilitated clarity enhancements. Content validity was established through expert consultation, and the questionnaire’s reliability was assessed using Cronbach’s alpha. The validated questionnaire was subsequently employed in the survey to accurately gauge participants’ knowledge of total knee arthroplasty.

The knowledge about total knee arthroplasty was assessed using a 19-item questionnaire where “true” was coded as 1 and “false” as 0 for response options. Negative questions were reverse-coded. The total knowledge score was calculated by summing all 19 items, with a potential range of 0 to 19 points. A higher score indicated a greater knowledge of total knee arthroplasty. Participants were classified based on their level of knowledge: poor if less than 50%, moderate if between 50% and 75%, and good if 75% or greater.

Statistical analysis

Categorical variables were given as frequency and percentages, while continuous variables were calculated and described as mean and standard deviation. The association between the knowledge score according to the sociodemographic characteristics and general awareness about total knee arthroplasty has been performed using the Mann-Whitney U test. A significant level was established at an alpha level of 0.05. All data analyses were tabulated and analyzed using IBM SPSS Statistics for Windows, Version 26 (Released 2019; IBM Corp., Armonk, New York, United States).

Ethical considerations

Ethical clearance for this study was obtained from the Ethics Committee of the Medical College at King Faisal University (No. 2023-2-611). All participants were informed of the study’s objectives, and their participation was voluntary. The questionnaire included a consent form that participants had to read and accept before answering the questions. The data collected were kept confidential, and only the research team could access them. The participants’ privacy and anonymity were maintained throughout the study, and their names and other personal identifiers were not recorded. Any data collected were only used for research purposes and were not shared with any other individual or organization.

## Results

Participant characteristics

In total, 704 participants completed the survey. Table 1 presents the sociodemographic characteristics of participants. The most frequent age group was 18 to 25 years (53.1%), with males constituting the majority (60.9%). Nearly 60% were single, and more than half (55.3%) held bachelor’s degrees. Additionally, 46.4% reported monthly earnings of less than 3,000 Saudi Riyals.

**Table 1:** Demographic characteristics of participants SAR: Saudi Riyal

Variable		Frequency	Percentage
Age group	18–25 years	374	53.1
	26–35 years	116	16.5
	36–50 years	115	16.3
	>50 years	99	14.1
Gender	Male	429	60.9
	Female	275	39.1
Marital status	Single	416	59.1
	Married	288	40.9
Educational level	Uneducated	4	0.6
	Primary school	10	01.4
	Intermediate school	17	02.4
	High school	171	24.3
	Diploma	73	10.4
	Bachelor degree	389	55.3
	PhD	40	05.7
Monthly income (SAR)	<3,000	327	46.4
	3,000–7,000	119	16.9
	7,001–12,000	76	10.8
	12,001–18,000	100	14.2
	18,001–25,000	82	11.6

Awareness and knowledge about total knee arthroplasty

In Table 2, 41.1% of participants reported knowing someone who had undergone or was going to undergo total knee arthroplasty. The prevalence of participants who had heard of total knee arthroplasty was 70.9%, and 39.5% had received information about total knee arthroplasty. Furthermore, 37.4% were aware of the indications and signs of total knee arthroplasty, and 36.2% believed that total knee arthroplasty is common in Saudi Arabia. Notably, 32.2% of participants thought that total knee arthroplasty is more commonly performed in men than in women.

**Table 2:** Awareness and perceptions of total knee arthroplasty

Statement	Response	Frequency	Percentage
Do you know anyone who did/will do total knee arthroplasty?	Yes	289	41.1
	No	415	58.9
Have you heard of total knee arthroplasty?	Yes	499	70.9
	No	205	29.1
Have you had any information about total knee arthroplasty?	Yes	278	39.5
	No	426	60.5
Are you aware of the signs and symptoms of the indication for total knee arthroplasty?	Yes	263	37.4
	No	441	62.6
Do you think that total knee arthroplasty is common in Saudi Arabia?	Yes	255	36.2
	No	117	16.6
	I don’t know	332	47.2
Do you think that total knee arthroplasty is more common among males than females?	Yes	227	32.2
	No	162	23.0
	I don’t know	315	44.7

Assessment of knowledge about pain management

The overall mean knowledge score was 6.61 ± 4.06, with 74.1% of participants demonstrating poor knowledge, 24.4% moderate knowledge, and 1.4% good knowledge levels. Table 3 provides a comprehensive insight into the participants’ understanding of total knee arthroplasty across several key domains. Notably, a significant proportion, 74.3%, correctly recognized that physical therapy is the most effective approach for managing chronic knee pain. Moreover, 78.6% acknowledged that pain is a valid indication for total knee arthroplasty, and 76.0% understood that anterior cruciate ligament or meniscus tears are not the primary reasons for undergoing total knee arthroplasty. However, a common misconception exists, with 39.6% believing that any knee pain can only be treated with total knee arthroplasty. These findings highlight the necessity for tailored educational interventions to enhance awareness and dispel misperceptions about total knee arthroplasty among the surveyed participants. In the realm of risk factors and indications, it was also noted that 71.7% recognized knee fractures as a significant reason for undergoing total knee arthroplasty, and 73.6% believed that after total knee arthroplasty, patients can typically pray without restrictions. Furthermore, a substantial portion, 79.8%, understood that individuals who have undergone total knee arthroplasty may need to take medications, such as painkillers and antibiotics, for an extended period.

**Table 3:** Participants’ understanding of total knee arthroplasty

Category	Statement	Correct response	
		Frequency	Percentage
Knowledge about pain management	Any knee pain can only be treated with total knee arthroplasty.	279	39.6
	The best approach to treating chronic knee pain is through the use of painkillers and injections.	366	51.9
	total knee arthroplasty is the preferred treatment choice for chronic knee pain that does not respond to other methods.	326	46.3
	The most effective method for addressing chronic knee pain is physical therapy.	523	74.3
Knowledge about risk factors and indications	The total knee arthroplasty is influenced by a patient’s age.	300	42.6
	The primary reason for undergoing total knee arthroplasty is knee arthritis.	213	30.3
	total knee arthroplasty is influenced by hereditary factors.	496	70.5
	The main reason for undergoing total knee arthroplasty is knee fracture.	505	71.7
	It is advisable to wait until the pain becomes unbearable before considering surgery.	184	26.1
	Knee anterior cruciate ligament or meniscus tears are not the primary reasons for doing total knee arthroplasty.	535	76.0
	Pain is an indication of total knee arthroplasty.	553	78.6
	Decreased range of motion is an indication of total knee arthroplasty.	609	86.5
Knowledge about prognosis	Patients require a rehabilitation program after the surgery.	462	65.6
	The typical outcome of the surgery is not usually disappointing.	396	56.2
	total knee arthroplasty restores normal knee function.	293	41.6
	The knees of individuals who have undergone the surgery can have a normal appearance.	291	41.3
	After undergoing the surgery, patients can typically pray without restrictions.	518	73.6
	People who have undergone the surgery usually experience a significant reduction in pain.	522	74.1
	People who have undergone the surgery may need to take medications, such as painkillers and antibiotics, for an extended period.	562	79.8

Association of knowledge with sociodemographic characteristics

Table 4 illustrates the differences in knowledge scores based on sociodemographic characteristics and general awareness of total knee arthroplasty. A higher knowledge score was found to be associated with being older (p=0.02), having a higher level of education (p=0.02), a better monthly income (p<0.01), knowing someone who will undergo or has undergone total knee arthroplasty (p<0.01), having heard of total knee arthroplasty (p<0.01), receiving total knee arthroplasty information (p<0.01), and awareness of the indications and signs of total knee arthroplasty (p<0.01).

**Table 4:** Participants’ mean knowledge score by demographic characteristics SAR: Saudi Riyal

Variable		Mean knowledge score	p-value
Age group	≤25 years	6.27 ± 4.04	0.02
	>25 years	6.99 ± 4.05	
Gender	Male	6.55 ± 4.13	0.71
	Female	6.69 ± 3.95	
Marital status	Single	6.40 ± 3.96	0.08
	Married	6.91 ± 4.19	
Educational level	Diploma or below	6.19 ± 4.08	0.02
	Bachelor or higher	6.88 ± 4.03	
Monthly income (SAR)	≤7,000	5.97 ± 4.14	<0.01
	>7,000	7.72 ± 3.67	
Do you know anyone who did/will do total knee arthroplasty?	Yes	7.57 ± 3.92	<0.01
	No	5.94 ± 4.03	
Have you heard of total knee arthroplasty?	Yes	7.46 ± 3.78	<0.01
	No	4.54 ± 3.97	
Have you had any information about total knee arthroplasty?	Yes	7.97 ± 3.94	<0.01
	No	5.72 ± 3.89	
Are you aware of the signs and symptoms of the indication for total knee arthroplasty?	Yes	7.89 ± 3.91	<0.01
	No	5.84 ± 3.96	
Do you think that total knee arthroplasty is common in Saudi Arabia?	Yes	8.39 ± 3.36	0.06
	No	7.44 ± 3.75	
Do you think that total knee arthroplasty is more common among males than females?	Yes	8.12 ± 3.15	0.85
	No	8.11 ± 3.70	

## Discussion

Awareness among the public about total knee arthroplasty is pivotal, as it directly influences individuals’ decision-making regarding the most suitable treatment for knee conditions. This awareness plays a crucial role in enabling patients to make informed choices about their healthcare [12-14]. This study offers valuable insights into the perception of total knee arthroplasty within the premises of Al-Ahsa, Saudi Arabia.

The study results highlight a significant lack of knowledge among the population concerning total knee arthroplasty. The mean knowledge score, on a scale from 0 to 19 points, was merely 6.61, with approximately 74.1% of participants exhibiting a poor level of knowledge. These findings closely mirror those of Al-Mohrej et al., where 71.4% of the sample population displayed inadequate knowledge about total knee arthroplasty [1]. Another study by Al-Rumaih et al. reported that 69.1% of adult Saudis had insufficient knowledge about joint replacement surgery [15].

Our study identified several factors influencing knowledge, such as age, education, income, personal connections to individuals who underwent total knee arthroplasty, familiarity with total knee arthroplasty, access to total knee arthroplasty information, and awareness of total knee arthroplasty. These results align with Al-Mohrej et al., where age, higher income, and personal connections to surgery patients positively impacted knowledge scores [1]. Kwoh et al. also supported these findings, highlighting the link between knowledge of total knee replacement and the willingness to undergo total knee arthroplasty [11]. However, a study by Alshammari et al. [16] identified education as the sole predictor of awareness, with no significant variation in awareness scores related to gender, nationality, residence, or a history of chronic diseases. Our study similarly revealed that gender, marital status, knowledge about the prevalence of total knee arthroplasty in Saudi Arabia, and the belief that total knee arthroplasty is more common in males than females were not significantly associated with knowledge scores.

In a study conducted among the general population in Riyadh, most respondents were aware of total knee arthroplasty (71.6%), yet 70.3% reported a lack of clear information about the surgery. This aligns with our results, where 70.9% of the population had heard of total knee arthroplasty; however, 39.5% indicated they had not received any information about the surgical intervention [16].

One limitation of this study is its reliance on an online survey, potentially introducing selection bias by excluding individuals without internet access. The study’s geographical limitation to Al-Ahsa, Saudi Arabia, might not fully represent the diversity of knowledge and attitudes toward total knee arthroplasty in the entire country. Additionally, the self-reported survey responses could be subject to recall bias and social desirability bias, potentially impacting data accuracy. The cross-sectional nature of the study precludes establishing causal relationships, emphasizing the need for longitudinal research to better understand changes in public knowledge and attitudes over time.

## Conclusions

In conclusion, this study reveals a significant gap in the public’s knowledge and awareness of total knee arthroplasty in Al-Ahsa, Saudi Arabia. With the majority of the population exhibiting limited knowledge, there is a pressing need for targeted patient education initiatives to dispel misconceptions and provide accurate information about total knee arthroplasty. Addressing these knowledge gaps is vital for promoting informed healthcare decision-making and achieving patient-centered care. Policymakers, healthcare providers, and public health authorities should collaborate to develop region-specific strategies aimed at enhancing public understanding of total knee arthroplasty, ultimately improving healthcare outcomes and reducing the burden on healthcare systems.

## Appendices

Survey on Knowledge and Attitudes Regarding Total Knee Arthroplasty (TKA) among Al-Hasa's Residents, Saudi Arabia

Participation Agreement:

Before we begin, please indicate your willingness to participate by selecting either "Yes" or "No" below.

-        Yes

-        No

Demographic Information:

To better understand the knowledge and attitudes of our respondents, we kindly request that you provide us with some basic demographic information. Please choose the options that best describe you.

o   Gender:

§  Male

§  Female

o   Marital Status:

§   Married

§   Single

o   Age:

§  18-25

§  26-35

§  36-50

§  Over 50

o   Education:

§  Uneducated

§  Primary school

§  Intermediate school

§  High school

§  Diploma

§  Bachelor's degree

§  Master's degree

§  PhD

o   Monthly Income:

§  Less than 3000

§  3000 to 7000

§  7000 to 12,000

§  12,000 to 18,000

§  Over 18,000

Awareness of TKA:

We are interested in your awareness of TKA, a surgical procedure to replace a damaged knee joint with an artificial one. Please answer the following questions based on your knowledge and experience.

o   Do you know anyone who has had or will have TKA?

§  No

§  Yes

Knowledge:

We aim to assess your knowledge about TKA. Please answer the following questions based on your awareness and understanding.

o   Have you heard of TKA?

§  Yes

§  No

o   Have you received any information about TKA?

§  Yes

§  No

o   Are you familiar with the signs and symptoms that indicate the need for TKA?

§  Yes

§  No

o   Do you believe TKA is a common procedure in Saudi Arabia?

§  Yes

§  No

§  I don't know

o   Do you believe TKA is more common among males than females?

§  Yes

§  No

§  I don't know

Pain Management:

TKA is often considered a treatment option for knee pain. Please indicate your beliefs and knowledge regarding knee pain management and TKA by selecting the response that best represents your view.

o   Is TKA the only treatment option for knee pain?

§  True

§  False

§  I don't know

o   Is TKA the preferred treatment for chronic knee pain that doesn't respond to other treatments?

§  True

§  False

§  I don't know

o   Is physical therapy the best approach for treating chronic knee pain?

§  True

§  False

§  I don't know

o   Are painkillers and injections the preferred method for managing chronic knee pain?

§  True

§  False

§  I don't know

Risk Factors and Indications:

In this section, we are interested in your views on the factors and indications related to TKA. Please answer the questions based on your beliefs and understanding.

o   Is pain a valid indication for TKA?

§  True

§  False

§  I don't know

o   Is a decreased range of motion a valid indication for TKA?

§  True

§  False

§  I don't know

o   Is the suitability of TKA influenced by a patient's age?

§  True

§  False

§  I don't know

o   Is TKA influenced by hereditary factors?

§  True

§  False

§  I don't know

o   Is it advisable to wait until the pain becomes unbearable before opting for surgery?

§  True

§  False

§  I don't know

o   What, in your opinion, is the most common reason for undergoing TKA?

§  Knee arthritis

§  Knee ACL or meniscus tears

§  Knee fracture

§  Other (please specify)

Prognosis:

TKA can have various outcomes and effects. Please share your opinions on the potential results and experiences associated with TKA by selecting the response that aligns with your beliefs.

o   Does TKA typically restore normal knee functions?

§  True

§  False

§  I don't know

o   Do individuals who undergo the surgery generally experience reduced pain quickly?

§  True

§  False

§  I don't know

o   Do the knees of those who have undergone the surgery usually regain a normal appearance?

§  True

§  False

§  I don't know

o   Is the outcome of TKA generally disappointing?

§  True

§  False

§  I don't know

o   Do patients typically require a post-surgery rehabilitation program?

§  True

§  False

§  I don't know

o   Do individuals who have had TKA usually need to take long-term medications like painkillers and antibiotics?

§  True

§  False

§  I don't know

o   Can patients pray normally after undergoing TKA?

§  True

§  False

§  I don't know
